# CRISPR-based assay reveals SARS-CoV-2 RNA dynamic changes and redistribution patterns in non-human primate model

**DOI:** 10.1080/22221751.2022.2038020

**Published:** 2022-02-21

**Authors:** Zhen Huang, Lili Zhang, Christopher J. Lyon, Bo Ning, Brady M. Youngquist, Alex Niu, Brandon J. Beddingfield, Nicholas J. Maness, Nakhle S. Saba, Chen-Zhong Li, Chad J. Roy, Tony Y. Hu

**Affiliations:** a Center for Cellular and Molecular Diagnostics, Tulane University School of Medicine, New Orleans, LA, USA; b State Key Laboratory of Food Science and Technology, Nanchang University, Nanchang, People’s Republic of China; c Department of Biochemistry and Molecular Biology, Tulane University School of Medicine, New Orleans, LA, USA; d Section of Hematology and Medical Oncology, Tulane University School of Medicine, New Orleans, LA, USA; e Division of Microbiology, Tulane National Primate Research Center, Covington, LA, USA; f Department of Microbiology & Immunology, Tulane University School of Medicine, New Orleans, LA, USA

**Keywords:** SARS-CoV-2, non-human primate, CRISPR, RT-qPCR, multi-site specimens

## Abstract

Mounting evidence indicates that SARS-CoV-2 can infect multiple systemic tissues, but few studies have evaluated SARS-CoV-2 RNA dynamics in multiple specimen types due to their reduced accessibility and diminished performance of RT-qPCR with non-respiratory specimens. Here, we employed an ultrasensitive CRISPR-RT–PCR assay to analyze longitudinal mucosal (nasal, buccal, pharyngeal, and rectal), plasma, and breath samples from SARS-CoV-2-infected non-human primates (NHPs) to detect dynamic changes in SARS-CoV-2 RNA level and distribution among these specimens. We observed that CRISPR-RT–PCR results consistently detected SARS-CoV-2 RNA in all sample types at most time points post-infection, and that SARS-CoV-2 infection dose and administration route did not markedly affect the CRISPR-RT–PCR signal detected in most specimen types. However, consistent RT-qPCR positive results were restricted to nasal, pharyngeal, and rectal swab samples, and tended to decrease earlier than CRISPR-RT–PCR results, reflecting lower assay sensitivity. SARS-CoV-2 RNA was detectable in both pulmonary and extrapulmonary specimens from early to late infection by CRISPR-RT–PCR, albeit with different abundance and kinetics, with SARS-CoV-2 RNA increases detected in plasma and rectal samples trailing those detected in upper respiratory tract samples. CRISPR-RT–PCR assays for SARS-CoV-2 RNA in non-respiratory specimens may thus permit direct diagnosis of suspected COVID-19 cases missed by RT–PCR, while tracking SARS-CoV-2 RNA in minimally invasive alternate specimens may better evaluate the progression and resolution of SARS-CoV-2 infections.

## Introduction

RT-qPCR assays that detect nasal swab SARS-CoV-2 RNA are the gold-standard for COVID-19 diagnosis, and have been used to analyze other specimen types with variable results [1,2]. Nasal tissue is an initial infection site and nasal swabs are convenient diagnostic specimens, but their results can be affected by sampling technique and time from virus exposure [3,4]. SARS-CoV-2 replication persists longer in lower vs. upper respiratory tract samples [5], indicating that nasal clearance does not necessarily reflect systemic clearance. Nasal swab results may also be inconsistent and produce false-negatives, with some COVID-19 cases reported to produce positive nasal swabs after two consecutive negative swabs [6]. Saliva, nasopharyngeal, pharyngeal, buccal swabs, and other upper respiratory tract samples exhibit similar limitations. Lower respiratory tract specimens demonstrate greater diagnostic sensitivity [1,2] but are not suitable for screening efforts. SARS-CoV-2 RNA is also detectable in rectal swab and blood samples [7,8], and its blood levels can predict COVID-19 severity and mortality [8], but low viral RNA concentrations in these sample are difficult to detect by RT-qPCR [1,2].

Few studies have evaluated SARS-CoV-2 RNA dynamics in multiple specimen types, and those that have done so have limitations that reduce their utility to evaluate systemic progression of SARS-CoV-2 infections. RT-qPCR assays used in these studies tend to exhibit insensitive and variable performance with non-respiratory tract specimens [1,2], sometimes yielding results contradicted by subsequent studies [9,10]. Most of these studies also did not obtain serial paired specimens, and relied on aggregate comparisons using specimens collected from different cases at different times after symptom onset. Most studies also relied on symptom development to estimate time since virus exposure, although SARS-CoV-2 exhibits variable latency [11].

Non-human primate (NHP) COVID-19 models can mimic human infection and permit highly controlled infection and sample procedures to address human study limitations [12–14], but remain limited by the ability of RT-qPCR to detect low concentration viral RNA in non-respiratory sample types. New assays employing clustered regularly interspaced palindromic repeat (CRISPR) complex activity to sequence-specifically enhance target nucleic acid signal can permit ultrasensitive disease diagnosis, including COVID-19 diagnosis [15]. Such assays employ the trans cleavage activity of a Cas12/gRNA complex bound to an amplicon recognition sequence to cleave and derepress a quenched fluorescent oligonucleotide reporter that is present at high concentration. This results in signal amplification versus standard RT-qPCR assays since multiple reporter oligonucleotides can be cleaved by a Cas12/gRNA complex bound to its target amplicon, whereas signal in conventional RT-qPCR assays results from a one-to-one binding of a reporter oligonucleotide with its target amplicon. CRISPR-based assays can detect single-copy targets [16] and distinguish targets with single base differences [17] to permit sensitive and accurate mapping of SARS-CoV-2 RNA changes in respiratory and non-respiratory samples, which could be used to evaluate prognosis, inform treatment decisions, and evaluate systemic clearance. We have reported that a CRISPR-based assay can detect SARS-CoV-2 RNA with approximately 5-fold greater sensitivity than RT-qPCR to detect viral RNA in plasma samples that produce false negatives when analyzed by RT-qPCR [10]. We therefore employed RT-qPCR and an ultrasensitive CRISPR fluorescence detection system (CRISPR-FDS) [18] to analyze viral RNA kinetics in longitudinal respiratory and non-respiratory specimens of NHPs after SARS-COV-2 infection. RT-qPCR and CRISPR-FDS results were similar for nasal and pharyngeal swabs, the only samples where RT-qPCR consistently detected longitudinal signal. CRISPR-FDS detected correlated signal in all respiratory samples, which preceded weaker but more durable correlated signal in non-respiratory samples. CRISPR-FDS may thus provide a means to evaluate systemic viral/viral RNA burden not detectable by RT-qPCR to improve diagnosis and better predict outcomes for SARS-CoV-2 infections.

## Materials and methods

### Key reagents

SuperScript IV One-Step RT–PCR System (catalog 1235820) and nuclease-free water (catalog 4387936) were purchased from Thermo Fisher Scientific. EnGen Lba Cas12a (catalog M0653T) and NEBuffer 2.1 (catalog B7202S) were purchased from New England Biolabs. Primers, gRNA, probes (Supplemental Table 1), and the CDC 2019-novel coronavirus (2019-nCoV) real-time RT–PCR diagnosis panel was synthesized by Integrated DNA Technologies (catalog 10006713).

### NHP experimental SARS-CoV-2 infection

The Institutional Animal Care and Use Committee of Tulane University reviewed and approved all procedures for NHP experiments in this study. The Tulane National Primate Research Center is fully accredited by the Association for Assessment and Accreditation of Laboratory Animal Care, and all animals receive care according to standards outlined in the NIH Guide to Laboratory Animal Care. The Tulane Institutional Biosafety Committee approved the procedures employed for sample handling, inactivation, and removal from BSL3 containment.

Sixteen adult male NHPs [8 African green monkeys (AGMs) and 8 Indian Rhesus macaques (RMs), Supplemental Table 2] were subjected to aerosol or multi-route mucosal SARS-C0V-2 exposure to mimic major routes of human infection. Four AGM and four RM were exposed to inhaled doses of aerosolized SARS-CoV-2 (BEI, USA-WA1/2020, NR-52281) of 5 × 10^3^ TCID50 and 1 × 10^4^ TCID_50_, respectively. Four AGM and four RM were exposed to a cumulative 1.2 × 10^6^ TCID_50_ dose via conjunctival, nasal, pharyngeal, and intratracheal routes. All animals were evaluated twice daily for 28 days-post-infection (dpi) by veterinary staff (Supplemental Table 3). Plasma and breath samples were collected from all animals 7 days before SARS-CoV-2 exposure and 1, 7, 14, 21, and 28 dpi. Nasal, buccal, pharyngeal, and rectal swabs were collected at all time points except 21 dpi (Supplemental Table 4).

### Clinical information and data collection

Human nasal swab, saliva, and plasma samples analyzed this study were collected from adult patients (*n* = 6) with a history of leukemia who presented with symptoms consistent with COVID-19 at Tulane Hospital from April 2020 to July 2020. Clinical samples and corresponding clinical data were collected for analysis under a general research use consent or after obtaining written informed consent from patients, who also indicated their assent, in compliance with a consent protocol (2020-595) by approved by the institutional review board of Tulane University.

### Sample collection and processing

All NHP samples were collected from anesthetized NHPs by a veterinarian. Sterile cytology swabs were used to gently scrub specified nasal, buccal, pharyngeal, and rectal mucosa sites for 5–10 s; blood samples were collected by venipuncture into EDTA tubes and rapidly processed for plasma isolation; and exhaled breath samples were collected in dorsal recumbent NHPs over 5 min interval using a modified pediatric face mask fitted with a HEPA-filtered inspiration port and a sampler [19]. Exhaled respiratory aerosols were continuously collected under negative pressure through an attached impinger (AGI-30, Ace Glass) operating at 6 liters/min that contained 10 mL DMEM in the collection vessel, after which the impingement liquid was aseptically decanted and processed for RNA. All patient nasal samples were collected by trained nurse according to Interim Guidelines for Collecting and Handling of Clinical Specimens for COVID-19 testing [20]; blood samples were collected by venipuncture into EDTA tubes and rapidly processed for plasma isolation; and saliva was collected in sterile, screw cap containers without preservative. Swab samples were stored at 25°C in 200 μL of DNA/RNA Shield Reagent (Zymo Research, R110-50) and plasma and saliva samples were stored at −80°C prior to RNA extraction.

### RNA extraction

RNA was extracted from 100 μL of swab or breath samples stored in 200 μL DNA/RNA Shield, or 100 μL of plasma, using the Zymo Quick-DNA/RNA Viral Kit (D7020) in an enhanced BL2/BL3 space following an Institutional Biosafety Committee-approved protocol, and stored at −80°C until analysis.

### CRISPR-FDS assay

The CRISPR-FDS assay targeting SARS-CoV-2 specific ORF1ab gene was performed in two steps. First, 5 μL RNA sample, 10 μL 2× Platinum SuperFi RT–PCR Master Mix, 0.2 μL SuperScript IV RT Mix, 2.8 μL nuclease-free water, and 1 μL each of 10 μM forward and reverse primers were incubated at 55°C for 10 min, and then subjected to a standard PCR protocol (5 min at 98°C; 38× [10 s at 98°C, 10 s at 60°C, 15 s at 72°C]; and 5 min at 72°C). Completed PCR reactions were transferred to 96-well half-area plate wells, mixed with 10 μL of CRISPR reaction reagents (3 μL of 10× NEBuffer 2.1, 3 μL of 300 nM gRNA, 1 μL of 1 μM EnGen Lba Cas12a, 1.5 μL of 10 μM fluorescent probe, and 1.5 μL nuclease-free water), and incubated at 37°C for 20 min in the dark. Fluorescence signal was then excited at 495 nm and read at 520 nm using a SpectraMax i3x Multi-Mode Microplate Reader (Molecular Devices). The positive threshold was defined as the mean plus 3 times the standard deviation of the CRISPR-FDS signal detected in baseline samples.

### RT-qPCR assay

RT-qPCR reactions used the CDC 2019-novel coronavirus (2019-nCoV) real-time RT-qPCR diagnosis panel for SARS-CoV-2 N1 gene using 5 μL of isolated RNA, 1.5 μL of Combined Primer/Probe Mix, 5 μL of 4× TaqPath 1-Step RT–PCR Master Mix (Thermo Fisher Scientific), 8.5 μL nuclease-free water and a QuantStudio 6 Flex Real-Time PCR System (Thermo Fisher Scientific, catalog 4485691) programmed with the reaction conditions specified for this assay. Samples with Ct values <40 were defined as positive.

### Statistics

Mann–Whitney tests were used to compare SARS-CoV-2 RNA levels between species, infection doses, and administration routes for individual specimen types. Pearson correlation coefficients were used to evaluate correlations among longitudinal SARS-CoV-2 RNA levels in different specimens. All tests were 2-sided with alpha of 0.05. GraphPad Prism (9.1.2) was employed for statistical analyses and data visualization.

## Results

### Pathology findings in SARS-CoV-2-infected NHP models

**Figure 1.**
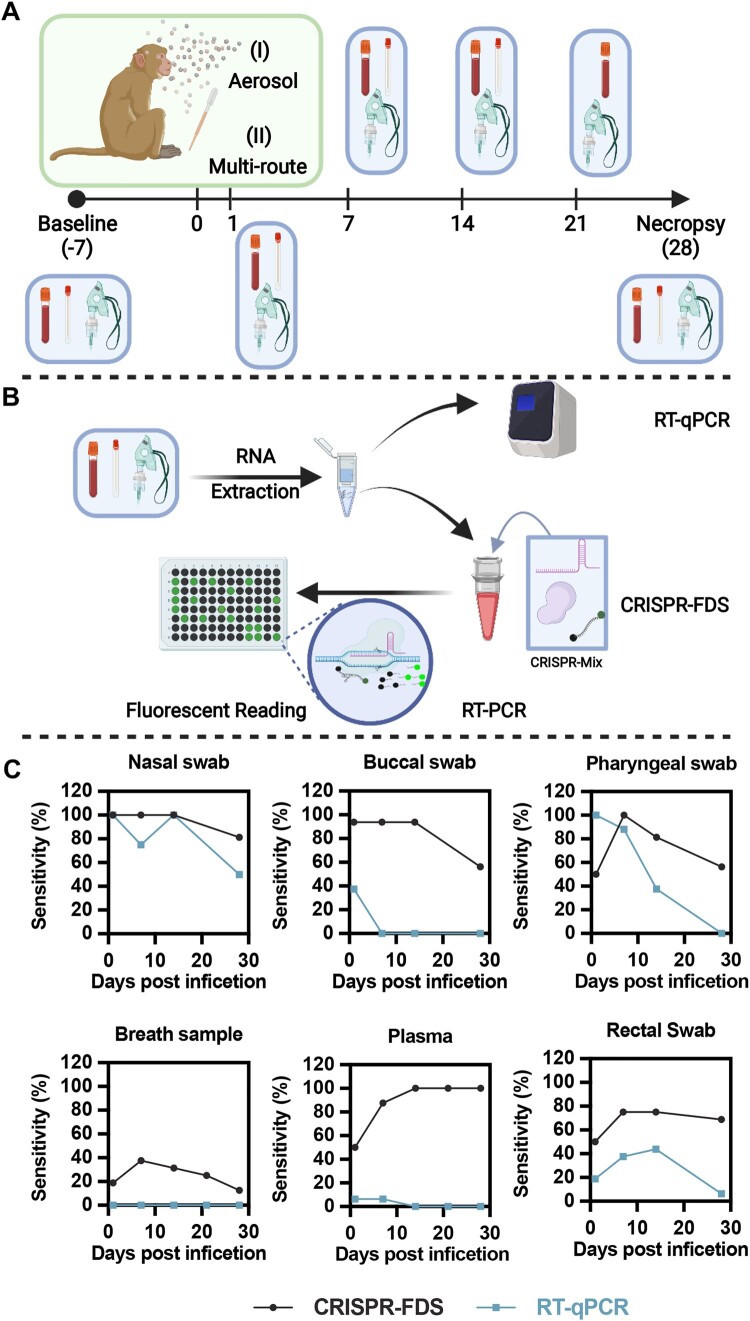
RT-qPCR and CRISPR-FDS detection of SARS-CoV-2 RNA in respiratory and non-respiratory samples of SARS-CoV-2-infected non-human primates (NHPs). (A) Schematic of the NHP study infection methods (aerosol and multi-route exposure), specimen types [mucosal (nasal, buccal, pharyngeal, and rectal) swab, blood, and breath samples], and their collection times relative to virus exposure. (B) Schematic of the RT-qPCR and CRISPR-FDS assay workflows. RNA extracted from NHP specimens added to RT-PCR reactions that were directly read by real-time PCR machine for RT-qPCR, or subjected to PCR and then supplemented with CRISPR-FDS assay reagents and read on a fluorescent plate reader. (C) RT-qPCR and CRISPR-FDS assay SARS-CoV-2 RNA diagnosis sensitivity in distinct specimen types at the indicated days post-infection (*N* = 16 samples/time point).

NHP models often closely mimic pathophysiologic responses associated with human disease and are useful in identifying and evaluating important mechanisms, therapeutic targets, and vaccine efficacy [14]. We therefore evaluated the ability of RT-qPCR and CRISPR-FDS to detect viral RNA in multiple specimens collected from four NHP models. Eight male RM aged 4–11 years and eight adult male AGM aged 7.5 years were infected with SARS-CoV-2 via different exposure routes (Figure 1A and Supplemental Table 2) for this study. Half the RMs and AGMs were exposed to aerosolized SARS-CoV-2 virus; and half were infected by a multi-route mucosal membrane exposure protocol for conjunctival, nasal, pharyngeal, and intratracheal membranes.

None of these NHPs revealed any overt signs or symptoms associated with SARS-CoV-2 infection, including fever, abnormal breathing, diarrhea, and weight loss, and gross post-mortem analysis of their respiratory tissue failed to detect major gross pathological abnormalities, consistent with results from previous studies [12–14]. These results agree with the emerging consensus that most NHP species emulate asymptomatic human infections, as a productive infection ensues post-exposure, but that there are few clinical signs that accompany an ultimately self-limiting disease [21]. Significant lung abnormalities were detected in all but one animal, but most lung pathology findings (4/5; emphysema, fibrinous pleuritis, multi-focal hemorrhage, pleuritis, lymphadenoma, and interstitial changes) were detected in aerosol-exposed RMs (Supplemental Table 3). Mild to moderate interstitial or perivascular inflammation was frequently observed, but alveolar septal thickening, focal fibrosis, and pleuritis was also detected, consistent with findings from other COVID-19 models [21]. No pathological differences were detected between any of the groups.

### CRISPR-FDS versus RT-qPCR sensitivity in paired longitudinal NHP samples

Mucosal (nasal, buccal, pharyngeal, and rectal) swab, blood, and breath samples collected pre- and post-infection were analyzed by RT-qPCR and CRISPR-FDS (Figure 1A-B and Supplemental Table 4). RT-qPCR and CRISPR-FDS both detected SARS-CoV-2 RNA in all nasal swabs at 1 dpi confirming virus exposure protocol effectiveness in both species, but exhibited different positive rates for 1 dpi buccal and pharyngeal swabs (Figure 1C and Supplemental Table 5). Both assays detected SARS-CoV-2 RNA in rectal swabs and plasma at 1 dpi, although positive rates were higher for CRISPR-FDS than RT-qPCR for both samples (50% and 50% vs. 18.8% and 6.3%), likely reflecting the higher limit of detection of RT-qPCR [10].

Both assays detected SARS-CoV-2 RNA in nasal swabs throughout the study, but their detection rates differentially decreased by 21–28 dpi, with RT-qPCR positive rates being more variable and falling more rapidly compared to CRISPR-FDS positives (Figure 1C). CRISPR-FDS nasal and buccal swab positive rates were similar until 14 dpi, after which buccal swabs more frequently positive. RT-qPCR, however, detected viral RNA in only about a third of buccal swabs at 1 dpi, after which all samples were tested negative.

CRISPR-FDS detection rate kinetics were similar in pharyngeal swab and breath samples (Figure 1C): low (50% and 19%) at 1 dpi, peaking at 7 dpi (100% and 38%) and declining thereafter (56% and 13% at 28 dpi). RT-qPCR, however, returned negatives results for all breath samples and it detection rate in pharyngeal swabs peaked at 1 dpi, remained high at 7 dpi (88%), and then markedly decreased at 14 dpi (38%), before falling to zero by 28 dpi. RT-qPCR and CRISPR-FDS results also markedly diverged for plasma, detecting positives in 6.3% and 50% of the 1 dpi samples, and zero versus all samples from 14 to 28 dpi (Figure 1C).

### Effect of NHP species, dose, and infection route on viral RNA level

Evaluation of SAR-CoV-2 RNA changes over time by RT-qPCR was possible only with nasal and pharyngeal swabs due to the scarcity of consistent positive results between NHP groups for other sample types (Supplemental Table 6), and compromised interpretation of pharyngeal RT-qPCR differences for one study group. Nasal and pharyngeal swab RT-qPCR signal tended to peak around 7 dpi, and their longitudinal values demonstrated good correlation but exhibited different positive rates for 1 dpi buccal and pharyngeal swabs (Supplemental Fig. 1A). RT-qPCR detected higher SARS-CoV-2 RNA levels in 7 dpi nasal swabs of NHPs subjected to high-dose multi-route versus low-dose aerosol virus exposure without considering species, apparently due to a differential AGM group response (Supplemental Fig. 1B-D). No differences were detected in the corresponding pharyngeal swabs, although RMs revealed higher 7 dpi RT-qPCR responses to multi-route versus aerosol virus exposure. No significant differences were detected between AGMs and RMs with or without regard to the virus exposure method (Supplemental Fig. 1E-G).

In contrast to RT-qPCR, CRISPR-FDS detected viral RNA in most longitudinal study specimens (mean 67%-95% positive), excepting only breath samples (mean 25% positive) (Supplemental Table 6). CRISPR-FDS-positive results were detected in more buccal swabs of NHPs infected by multi-route exposure, and more rectal swab and plasma samples of NHPs infected by aerosol exposure (Supplemental Table 6).

CRISPR-FDS signal tended to cluster by sample type. CRISPR-FDS signal in upper respiratory tract samples (nasal, buccal and pharyngeal swabs) tended to peak between 7 and 14 dpi, while a breath sample had fewer dynamic changes (Supplemental Fig. 2A-D). Rectal swab and plasma samples tended to reveal modest increases that appeared to plateau around 14 dpi and remain stable thereafter (Supplemental Fig. 2E-F). CRISPR-FDS signal in longitudinal respiratory tract and breath samples, and rectal swab and plasma samples, exhibited strong correlations (Supplemental Fig. 2G). CRISPR-FDS signal in buccal and pharyngeal swabs and breath samples correlated more strongly than with nasal swab signal (Pearson r-values ≥0.96 vs. 0.72–0.87). Similarly, rectal swab and plasma signal correlated more strongly with signal from buccal and pharyngeal swabs and breath samples than with nasal swab signal, although plasma correlations with nasal swab and other respiratory samples (Pearson correlation coefficient of 0.20 versus 0.64–0.79) tended to be stronger than matching rectal swab correlations (Pearson correlation coefficient of 0.14 versus 0.46–0.64).

**Figure 2.**
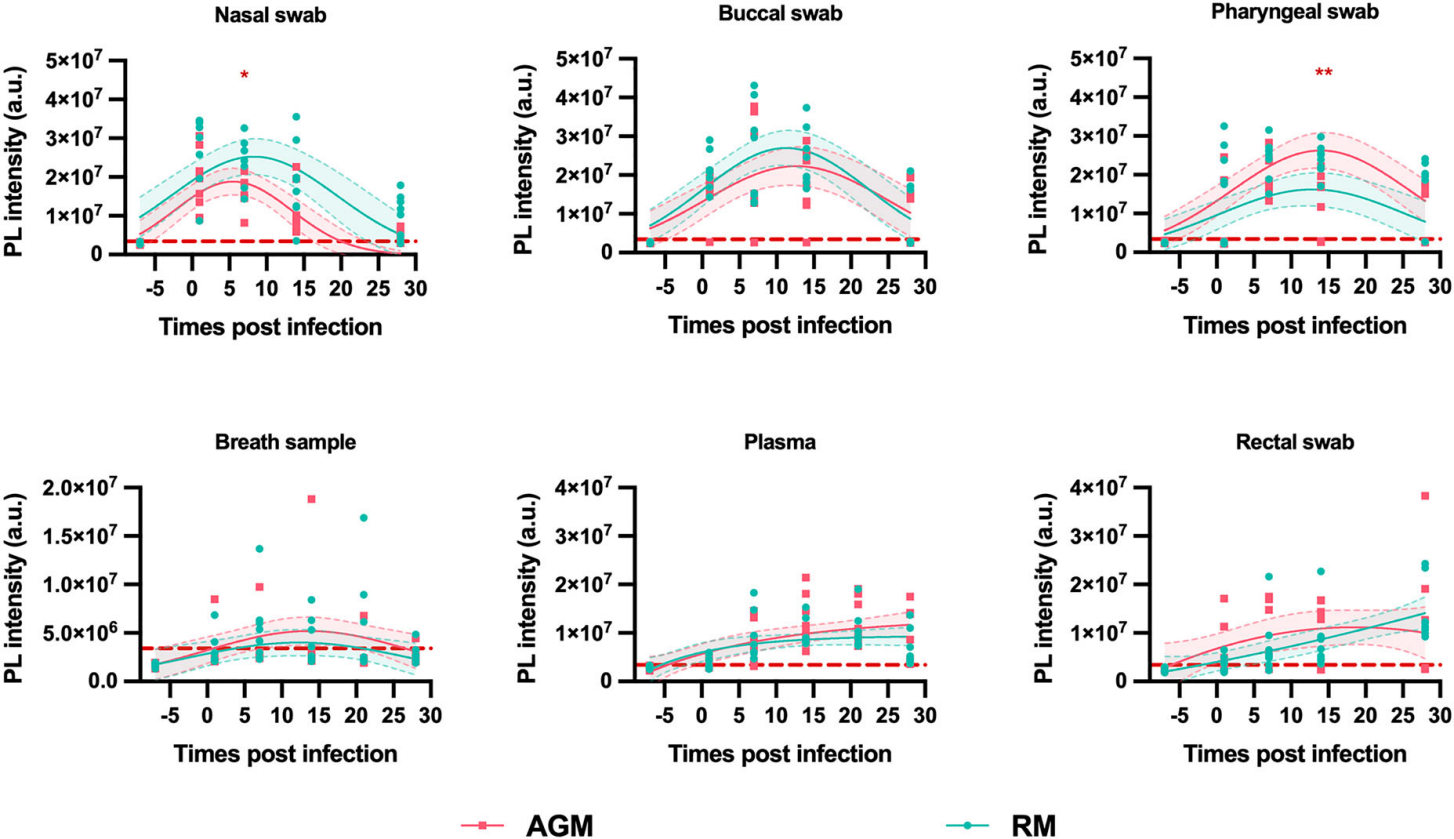
CRISPR-FDS SARS-CoV-2 RNA signal in different specimens of African green monkeys (AGMs, *n* = 8) and Indian Rhesus macaques (RM, *n* = 8) at the indicated days pre- and post-infection. Data points represent the mean of technical replicates for each sample. Blue and grey shaded regions and dashed lines indicate the 95% confidence intervals of the fitted lines. CRISPR-FDS assay signal is depicted as photoluminescent (PL) intensity presented in arbitrary units (a.u.) with the threshold for positive signal (3.4 × 10^6^ a.u.), as described in Methods, indicated by a dashed red line. (*, *p* < 0.05; **, *p* < 0.01 by Mann-Whitney test).

**Figure 3.**
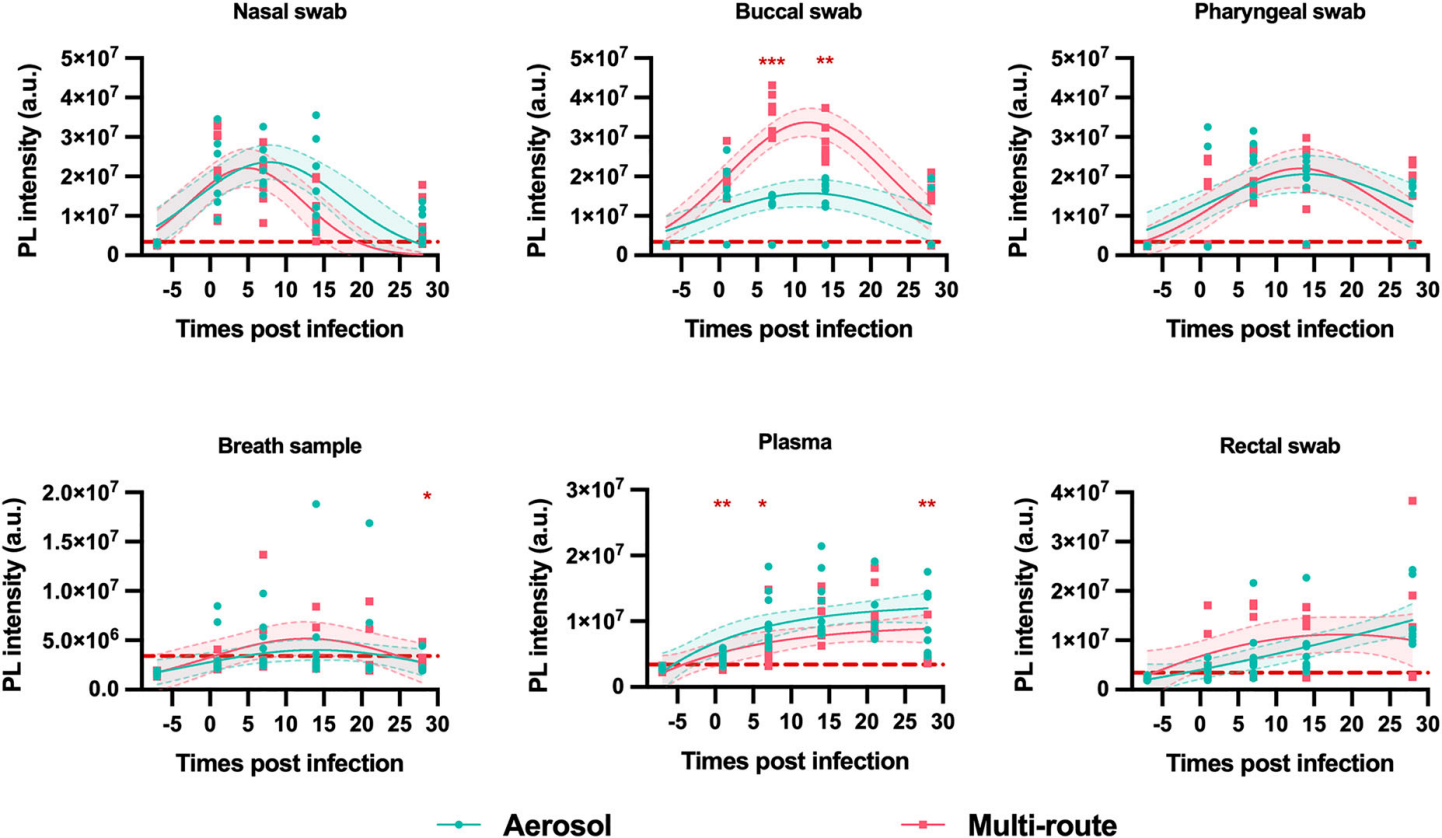
CRISPR-FDS SARS-CoV-2 RNA signal in different specimens of NHPs exposed to aerosol (*n* = 8) versus multi-route (*n* = 8) SARS-CoV-2 exposure. Data points represent the mean of technical replicates for each sample. Blue and grey shaded regions and dashed lines indicate the 95% confidence intervals of the fitted lines. CRISPR-FDS assay signal is depicted as photoluminescent (PL) intensity presented in arbitrary units (a.u.) with the threshold for positive signal (3.4 × 10^6^ a.u.), as described in Methods, indicated by a dashed red line. (*, *p* < 0.05; **, *p* < 0.01; ***, *p* < 0.001 by Mann-Whitney test).

No consistent species-specific differences were detected when comparing CRISPR-FDS signal in all sample types disregarding dose or exposure route (Figure 2), but CRISPR-FDS signal tended to be higher in AGM vs. RM nasal and pharyngeal swabs at most intervals. These differences achieved significance at different dpi for nasal and pharyngeal swabs (7 and 14 dpi, respectively) but not in other specimens. Major consistent differences related to dose/exposure route were not observed in most samples when disregarding species, with the exception of buccal swabs. NHPs subjected to high-dose/multi-route exposure had markedly higher buccal swab signal at 7–14 dpi, likely due to direct exposure of buccal tissue to high-dose virus during pharyngeal and intratracheal administration (Figure 3). CRISPR-FDS also detected moderate signal differences (1.3- to 2.4-fold) in plasma of NHPs subjected to low-dose aerosol versus high-dose multi-route infection (Figure 3), with aerosol-exposed NHPs exhibiting a more rapid signal response (88% versus 13% positive at 1 dpi).

CRISPR-FDS signal tended to be higher in all respiratory swabs obtained from AGM versus RM subjected to aerosol infection (Supplemental Fig. 3), and similar trends were observed in AGMs versus RMs infected *via* multi-route exposure (Supplemental Fig. 4). However, AGMs and RMs subjected to multi-route infection had positive pharyngeal swab signal at 1–28 and 1–14 dpi, respectively, with AGMs revealing greater signal from 7 to 28 dpi.

Comparison of aerosol versus multi-route exposure effects in AGM and RM groups, found that the multi-route infection markedly increased CRISPR-FDS signal in buccal swabs of both species (Supplemental Fig. 5–6). SARS-CoV-2 RNA kinetics tended to differ in the pharyngeal samples of these animals, with RNA signal declining earlier in RMs vs. AGMs subjected to multi-route virus exposure.

### Comparison of CRISPR-FDS results in patient samples

**Figure 4.**
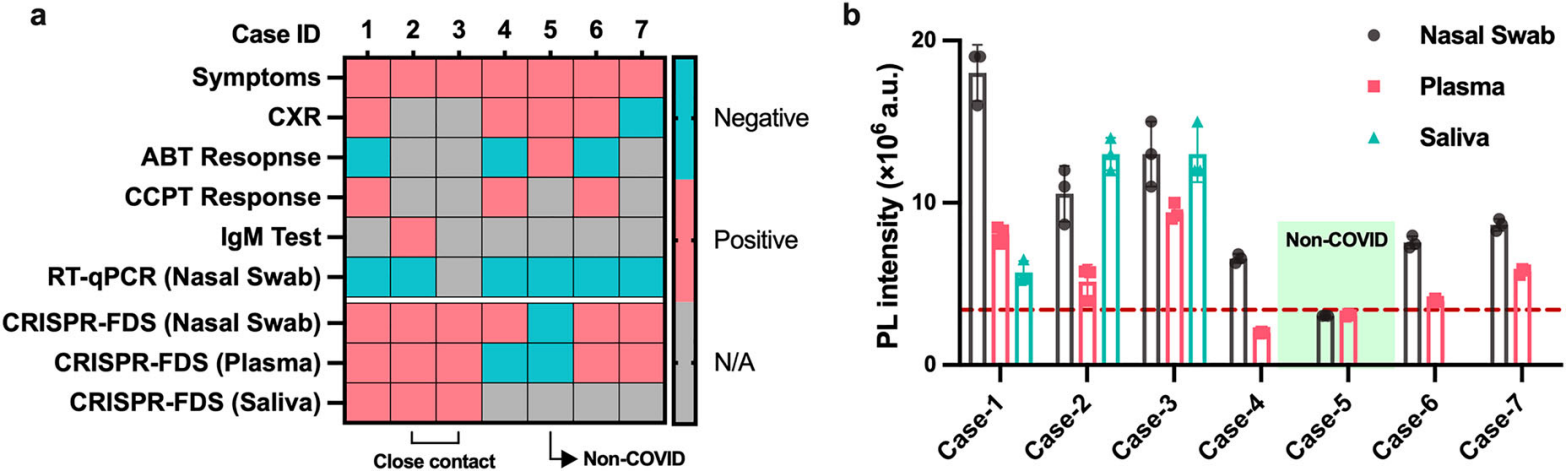
CRISPR-FDS evaluation of a small group of symptomatic adults with suspected COVID-19 cases who had negative or missing COVID-19 RT-qPCR results. (a) Clinical and assay data and (b) CRISPR-FDS SARS-CoV-2 RNA signal in paired nasal swab, plasma, and/or saliva samples obtained from or without positive nasal swab RT-qPCR results for COVID-19. Detailed case histories are presented in Figure S7–12. Results shown in (a) indicate clinical and assay results consistent with COVID-19, including positive chest X-ray (CXR) or SARS-CoV-2 IgM results, response to COVID-19 conditioned plasma therapy (CCPT), and negative response to antibiotic treatment (ABT). CRISPR-FDS assay signal is depicted as photoluminescent (PL) intensity presented in arbitrary units (a.u.) with the threshold for positive signal (3.4 × 10^6^ a.u.), as described in Methods, indicated by a dashed red line.

Similar analyses cannot be performed with COVID-19 patients, since initial exposure times are often unknown, and it is not practical to collect the same array of longitudinal samples. However, several trends could be observed following CRISPR-FDS analysis of samples available from a small group of COVID-19 cases, including three cases whose plasma SARS-CoV-2 RNA levels we have previously reported [10]. Most patients with evidence of SARS-CoV-2 infection had CRISPR-FDS-positive nasal swab and plasma results (83%; 5/6), although plasma signal tended to be lower (Figure 4 and Supplemental Figs. 7–12), similar to results observed in the NHP models, while a patient (Case-5) that received an alternate diagnosis due to response to antibiotic treatment had negative results for both samples.

These patients did not have buccal and pharyngeal swabs and thus could not be assessed for potential correlations among respiratory samples detected in the NHP models. Saliva CRISPR-FDS signal, measured as a potential alternate indicator of upper respiratory tract infection in a subset of patients, was not completely consistent with nasal swab signal, but both were CRISPR-FDS positive for all patients diagnosed with COVID-19.

## Discussion

COVID-19 is primarily diagnosed by RT-qPCR detection of SARS-CoV-2 RNA in swabs of the upper respiratory tract [22], but viral load in these specimens may vary with sample site and disease duration [4] to affect diagnostic accuracy. RT-qPCR analysis of lower respiratory tract specimens (e.g. bronchoalveolar lavage fluid) can yield greater diagnostic sensitivity (93%) than nasal (63%) and pharyngeal (32%) swab analyses. Sub-genomic SAR-CoV-2 RNA indicative of active infection can also be detected longer in lower versus upper respiratory tract infections, indicating the former may be more diagnostically useful [1], but the collection of these samples is impractical and not recommended for most suspected COVID-19 cases [7]. RT-qPCR false-negative rates for upper respiratory tract samples are approximately 38% at symptom onset (presumed 5 dpi), decrease to 20% at 8 dpi, and increase to 66% by 21 dpi, roughly paralleling RT-qPCR and CRISPR-FDS results for NHP nasal swabs in our study.

Longitudinal NHP lower respiratory tract specimens were not collected, however, preventing direct comparison of upper versus lower respiratory tract SARS-CoV-2 signal. CRISPR-FDS signal detected in plasma and rectal swabs persisted after it was reduced or absent in respiratory swabs, providing evidence for an extrapulmonary infection that likely spread from respiratory to gastro-intestinal tract tissue *via* the circulation. This was not detected by RT-qPCR, which provided only sporadic and highly variable results for these non-respiratory tract samples.

CRISPR-FDS but not RT-qPCR also detected SARS-CoV-2 RNA in individual NHP breath samples collected as late as 28 dpi, suggesting these asymptomatic animals might have been capable of transmitting SARS-CoV-2 *via* respiratory droplets, despite exhibiting negative RT-qPCR results in their corresponding upper respiratory tract samples. NHP breath samples were collected from sedated asymptomatic NHPs, however, and it is unclear how well these correspond to those of asymptomatic patients, since exertion and speech have been shown to greatly influence the production of respiratory droplets and aerosols implicated in airborne viral transmission [23].

SARS-CoV-2 RNAemia has been shown to predict disease severity [8,10,24,25], but RT-qPCR exhibits poor and variable sensitivity to detect SARS-CoV-2 RNA in plasma or serum from confirmed COVID-19 cases [1,26] and thus has very limited utility for COVID-19 diagnosis and prognosis. RT-qPCR also exhibited very poor diagnostic sensitivity in the current study, detecting SARS-CoV-2 RNAemia in only two NHP plasma samples, but positive plasma CRISPR-FDS signal was detected in all our NHPs, with positive samples detected as early as 1 dpi and persisting until 28 dpi. Notably, all NHPs in this study had asymptomatic SARS-CoV-2 infections and thus would be expected to have low RNAemia based on previous studies and our RT-qPCR results. Serum or plasma CRISPR-FDS results thus may be useful for COVID-19 diagnosis and prognosis across the full spectrum of SARS-CoV-2 infection, including asymptomatic patients and patients with long-term infections with negative RT-qPCR test results [10].

RT-qPCR did not detect longitudinal signal in most NHP samples, preventing most subgroup analyses, but robust longitudinal CRISPR-FDS results detected differences corresponding to NHP species or virus exposure route. AGMs tended to have moderately higher CRISPR-FDS signal in respiratory swabs when NHPs were grouped by species, disregarding virus exposure route. Subgroup analysis also emphasized a difference observed in the pharyngeal response to multi-route exposure, although a potential mechanism for this difference is not obvious. The altered CRISPR-FDS signal intensity and kinetics observed in NHPs subjected to aerosol versus multi-route exposure likely reflects more efficient lower respiratory tract penetration that may increase pulmonary infection severity and resulting tissue injury to promote viral RNA release into the circulation.

Positive CRISPR-FDS and RT-qPCR assay results observed in this study cannot distinguish SARS-CoV-2 RNA that is packaged in infectious virus particles or directly shed by infected cells. Most NHP COVID-19 models exhibit productive infections in most mucosal and respiratory tissues [12,13]. Median time from symptom onset to viral clearance was determined to be 10 days by viral culture assays but 34 days RT-qPCR [27]. Low levels of SARS-CoV-2 RNA can also be detected in the blood of COVID-19 patients and animal models [8,24], but infectious virus has not been isolated from these samples and blood is therefore considered a non-infectious specimen [28]. SARS-CoV-2 culture assays typically require relatively high concentrations of virus, thus it can be difficult to conclusively determine if low concentrations of viral RNA derive from shed virus or viral RNA.

Several factors may limit the interpretation of these study results. First, NHP subgroup sizes are small and there is no comparator assay that can be used to confirm the validity of positive CRISPR-FDS signal detected in these samples. Second, CRISPR-FDS results detected with these specimens reflect viral RNA levels present in NHPs with asymptomatic SARS-CoV-2 infections, and the kinetics and degree of virus abundance in these samples may differ in animals with more severe infections and pathology. Third, the NHP study was limited to 28 dpi, at which time SARS-CoV-2 RNA was still detected in most rectal swabs and all plasma samples, and it thus not clear how long viral RNA may remain detectable in these samples. Finally, we do not have access to large clinical cohorts with comparable symptoms, known exposure dates, and similar samples and collection intervals to the analyzed NHP groups, limiting our ability to directly compare NHP and patient results. Further studies using larger patient cohorts that more closely resemble the phenotype and sample collection protocol of the NHP COVID-19 models are therefore needed to evaluate how viral RNA abundance and kinetics observed in these groups compare to human responses.

Results presented in this study indicate that CRISPR-FDS signal can detect SARS-CoV-2 RNA in multiple NHP specimen types that test negative, or intermittently positive, when analyzed by RT-qPCR to allow improved understanding of the relative degree and kinetics of viral/viral RNA expression in respiratory and extrapulmonary tissue. Better understanding of these processes could provide new information about the infection process that could enhance the diagnosis of challenging COVID-19 cases, including asymptomatic and long-term infections; prognostic evaluation of newly diagnosed patients to improve risk assessment and treatment management strategies; and evaluation of viral clearance. However, this CRISPR-FDS assay requires large-scale clinical studies to validate its performance, and should be regarded as an auxiliary test to the gold-standard of RT-qPCR for COVID-19 diagnosis.

## Supplementary Material

Supplemental Material
