# Supplementary material for: CRISPR-based assay reveals SARS-CoV-2 RNA dynamic changes and redistribution patterns in non-human primate model
Source: Emerg Microbes Infect. 2022 Feb 21;11(1):629–38. doi: 10.1080/22221751.2022.2038020 (PMC8865122; doi:10.1080/22221751.2022.2038020)
Supplement: Supplemental Material [file TEMI_A_2038020_SM6933.docx]

**Supplemental Data for**

**CRISPR-based Assay Reveals SARS-CoV-2 RNA Dynamic Changes and Redistribution Patterns in Non-Human Primate Model**

Zhen Huang^1,2,3^*, Lili Zhang^1,3^*, Christopher J. Lyon^1,3^, Bo Ning^1,3^, Brady M. Youngquist^1,3^, Alex Niu^4^, Brandon J. Beddingfield^5^, Nicholas J. Maness^5,6^, Nakhle S. Saba^4^, Chen-Zhong Li^1,3^, Chad J. Roy^5,6^, and Tony Y. Hu^1,3†^

^1^Center for Cellular and Molecular Diagnostics, Tulane University School of Medicine, New Orleans, Louisiana, USA.

^2^State Key Laboratory of Food Science and Technology, Nanchang University, Nanchang, China.

^3^Department of Biochemistry and Molecular Biology, Tulane University School of Medicine, New Orleans, Louisiana, USA.

^4^Section of Hematology and Medical Oncology, Tulane University School of Medicine, New Orleans, Louisiana, USA.

^5^Division of Microbiology, Tulane National Primate Research Center, Covington, Louisiana, USA.

^6^Department of Microbiology & Immunology, Tulane University School of Medicine, New Orleans, Louisiana, USA.

* These authors contributed equally to this work.

† **Correspondence to Tony Y. Hu**: J. Bennett Johnston Building Rm 474, 1324 Tulane Ave, New Orleans, LA 70112. Tel: 504-988-5310. Email: tonyhu@tulane.edu

**Supplemental Table 1. Oligonucleotide sequences used in this study.**

| Oligonucleotide ID | Sequence | Target |
| --- | --- | --- |
| ORF1ab-F | CCCTGTGGGTTTTACACTTAA | ORF1ab |
| ORF1ab-R | ACGATTGTGCATCAGCTGA | ORF1ab |
| gRNA -ORF1ab | UAAUUUCUACUCUUGUAGAUCACAUACCGCAGACGGUACAGAC | ORF1ab |
| Probe | FAM-TTTTTTTTTTTT-BHQ |  |

Underlined sequence indicates the region complementary to ORF1b target sequences

**Supplemental Table 2. Study animal.**

| Animal ID | Age (years) | Sex | Species | Route of challenge | Virus exposure  （TCID_50_） |
| --- | --- | --- | --- | --- | --- |
| IJ01 | 11.15 | Male | RM | Aerosol | 1×10^4^ |
| IR12 | 10.85 | Male | RM | Aerosol | 1×10^4^ |
| JG28 | 10.18 | Male | RM | Aerosol | 1×10^4^ |
| KN90 | 7.15 | Male | RM | Aerosol | 1×10^4^ |
| NB76 | 7.53 | Male | AGM | Aerosol | 5×10^3^ |
| NB78 | 7.53 | Male | AGM | Aerosol | 5×10^3^ |
| NB86 | 7.53 | Male | AGM | Aerosol | 5×10^3^ |
| NC06 | 7.53 | Male | AGM | Aerosol | 5×10^3^ |
| JK41 | 9.21 | Male | RM | Multi-route | 1.2×10^6^ |
| KH77 | 7.93 | Male | RM | Multi-route | 1.2×10^6^ |
| KT88 | 6.99 | Male | RM | Multi-route | 1.2×10^6^ |
| LP05 | 3.99 | Male | RM | Multi-route | 1.2×10^6^ |
| NB77 | 7.55 | Male | AGM | Multi-route | 1.2×10^6^ |
| NB81 | 7.55 | Male | AGM | Multi-route | 1.2×10^6^ |
| NC04 | 7.55 | Male | AGM | Multi-route | 1.2×10^6^ |
| NC07 | 7.55 | Male | AGM | Multi-route | 1.2×10^6^ |

TCID_50_, 50% tissue culture infectious dose; RM, Indian Rhesus Macaques; AGM, African Green Monkey.

**Supplemental Table 3.** **Heterogeneous clinical and pathological outcomes of NHPs following SARS-CoV-2 infection.**

| An ID | Summary of Gross Findings | Case Summary |
| --- | --- | --- |
| IJ01 | Mild congestion and emphysema of the right lower and left anterior lung, respectively. | The lungs of this animal had minimal chronic interstitial inflammation with rare type II pneumocyte hyperplasia. Additionally, this animal had mild to moderate pneumoconiosis, evident adjacent to most airways and within the tracheobronchial lymph nodes. Inflammation in other organs/tissues was limited. Eosinophilic inflammation in the small and large intestine is likely due to intestinal parasitism, although a definitive cause was not observed. Lymphoid tissues often exhibited mild to moderate lymphoid hyperplasia. |
| IR12 | Mild fibrinous pleuritis | This animal had minimal to mild interstitial inflammation in the lung. There were rare foci of mineralization within bronchi and small focal areas of fibrosis. Additionally, this animal had chronic inflammation of the stomach and colon. In the cecum this was associated with low numbers of whipworms. The lymph nodes exhibited variable lymphoid hyperplasia and sinus histiocytosis. |
| JG28 | Multifocal pulmonary hemorrhage and generalized, moderate, lymph adenomegaly. | This animal had scattered interstitial and perivascular inflammation (mild) in both the left and right lungs. In some lobes infiltration of inflammatory cells into vessel walls is indicative of vasculitis (lymphocytic). Multinucleated cells and rare granulomas were seen in some lung sections. The presence of foreign material within airways indicates prior aspiration and confounds the interpretation of the other microscopic findings. Special stains would be needed to further investigate the underlying cause for the inflammation and vasculitis in this case. Vasculitis and my intimal proliferation were noted in multiple organs in tissues predominately in mesenteric vessels. The morphologic features of the vasculitis and distribution are most consistent with polyarteritis nodosa, but other causes including viral vasculitis would also have to be considered. Additionally, this animal had mild myocarditis, gastritis, typhlitis, and interstitial nephritis. Lymph nodes were often moderately to markedly hyperplastic. |
| KN90 | Lung: Chronic interstitial changes (likely pneumoconiosis) | This animal had no gross evidence of pneumonia. The lungs have a diffuse, "doughy" texture. There are pinpoint multifocal tan foci scattered throughout the lung lobes, predominately along the dorsal margin. The pleura showed moderate inflammation with fibrosis. Lymph nodes of the lungs showed moderate perivascular inflammation. Inflammation and hyperplasia of variable levels are found in lymph nodes of multiple sites. |
| NB76 | Widespread reddening of all lung lobes. Gross findings are compatible with congestion or euthanasia artifact. No lesions were noted within other tissues. | This animal was enrolled in a CoV-2 study. Several microscopic changes were noted within the lungs and airways that are interpreted as being artifactual. These include the extensive fluid accumulation in alveoli, interpreted because of sodium pentobarbital administration; and the segmental loss of epithelium in airways that is interpreted as an artifact of collection procedures due to the lack of inflammation associated with these lesions. Perivascular inflammation was rarely noted within the right lung which may be a result of viral infection. No significant pathologic lesions were noted within other organs. Minimal to mild scattered chronic inflammation was noted within the liver, kidney, pancreas, and sections of the gut. The lymphocytic inflammation at these sites is a nonspecific microscopic change and would require special stains (IHC/ISH) to investigate further. Lymphoid hyperplasia was noted within the cervical and mesenteric lymph node. Lymphoid hyperplasia can occur in response to viral infection as well as an assortment of antigenic stimuli |
| NB78 | The reddening of the lungs and BAL are interpreted as euthanasia artifact. | No significant microscopic abnormalities were noted. Small regions of the lungs exhibited mild increased alveolar septal thickness; however, inflammation was not evident. Fluid infiltration into the left lungs is interpreted as euthanasia artifact. Mild, scattered inflammation was noted within the trachea, kidney, and stomach. A cause for the inflammation in these tissues was not observed. |
| NB86 | No significant gross abnormalities | This animal had mild scattered lymphocytic interstitial inflammation and alveolar septal thickening. Type II pneumocyte hyperplasia was rarely observed. These findings could indicate previous viral pneumonia; however, more diagnostic features of SARS-CoV-2 like syncytial cells and atypical type II pneumocyte hyperplasia were not observed. Mild scattered lymphocytic inflammation was noted within the kidney, liver, and prostate; however, a cause for the inflammation was not observed. Several lymph nodes exhibit lymphoid hyperplasia and peripheral nodes had prominent neutrophilic infiltration at the corticomedullary junction. |
| NC06 | The mottling of the right lower lung is interpreted as a euthanasia artifact | This animal had minimal interstitial inflammation within the left lungs in association with mild pneumocyte hyperplasia. These findings could indicate prior viral infection, but the minimal severity precludes any definitive statements. Special stains (IHC and in situ) are recommended to further pursue a viral etiology for these changes. Attenuation and sloughing of epithelial cells within airways was noted without inflammation and is therefore interpreted as artifactual. Multiple lymph nodes had neutrophilic infiltration along the corticomedullary junction. The cause for finding could not be definitively determined, and in the absence of peripheral neutrophilia may be due to redistribution. Minimal inflammation was noted the kidney, prostate, and GI tract. The level of inflammation is consistent with background lesions commonly observed in animals on and off projects. |
| JK41 | Mottling of the lungs is presumed to be a euthanasia artifact. Chronic gastritis. | Mild interstitial and perivascular inflammation was noted within the lungs. The presence of bronchiole epithelial hyperplasia and rare foci of mineralization in some sections is suggestive of previous lung damage. In addition to the pulmonary changes, mild myocarditis and interstitial nephritis were observed. A cause for the inflammation within the heart and kidney was not apparent. Chronic active inflammation was noted within the stomach and large intestine, a cause for the inflammation was not observed. Lymphoid hyperplasia was only observed in the mesenteric and inguinal lymph nodes. |
| KH77 | Renal cortical cyst. Mild lymph adenomegaly of the submandibular lymph node. | This animal had moderate, chronic pleuritis with scattered minimal to mild interstitial inflammation within the lungs. A cause for the inflammation was not observed. Rare aggregates of histiocytes may indicate mild, previous bouts of aspiration. The loss of epithelium within the airways is believed to be artifactual, or a consequence of sampling procedures. Chronic inflammation was noted in the upper respiratory tract. Outside the respiratory system. There was scant inflammation in the pancreas, marked gastritis, and mild colitis. The gastrointestinal inflammation is presumed to be due to chronic bacterial infection. Perivascular dermatitis of unknown cause was also present. Rare giant cells were seen invading the basal layer of the epidermis. |
| KT88 | Multifocal pulmonary bullae. | The lungs exhibited minimal interstitial and perivascular inflammation. Similarly mild inflammation was noted within the upper respiratory tract (trachea, nasopharynx, and nasal turbinate). The stomach exhibited sever chronic, active inflammation with glandular abscesses suggestive of a bacterial gastritis. The remainder of the intestinal tract had no to minimal inflammation. There was minimal interstitial nephritis. No other significant abnormalities were noted. |
| LP05 | Pulmonary congestion and edema (possible euthanasia artifact). | This animal had minimal to mild interstitial inflammation with a small amount of foreign material in the left anterior lung lobe. Artifactual changes included edema (euthanasia) and loss of respiratory epithelium (sample collection) were also noted. Chronic inflammation was present in the upper respiratory tract including the nasal septum and nasopharynx. Inflammation outside the respiratory system included mild myocarditis, moderate gastritis, and mild enterocolitis. Lymphoid tissues exhibited mild to moderate lymphoid hyperplasia. |
| NB77 | Pulmonary edema of the left anterior and posterior lung lobes (possible euthanasia artifact) | There were no inflammatory lesions noted within the lungs of this animal. Proteinaceous fluid within alveoli of the left lung is interpreted as euthanasia artifact. Similarly, sloughing and attenuation of airway epithelium is interpreted as being artifactual. Other organs and tissues contained only minor changes. This included chronic inflammation in the heart, gallbladder, kidney, and colon. Lymphoid hyperplasia was present with the bronchial lymph node along with phagocytosed environmental particulates (presumed silica). |
| NB81 | No significant gross abnormalities. | Pulmonary changes included minimal multifocal interstitial inflammation. A definitive cause for the inflammation was not observed. The right lung contained a focal area of granulomatous inflammation surrounding foreign debris indicating previous aspiration. Changes within other organs were minimal and not specific. These included interstitial mineralization of the kidney, interpreted as dystrophic given the limited distribution. Peripheral lymph nodes exhibited neutrophilic infiltration at the corticomedullary junction, a cause for which was not apparent. |
| NC04 | No significant gross abnormalities | Minimal interstitial inflammation was present within the lungs. One pulmonary vessel contained a partially incorporated fibrinous thrombus. No evidence of thrombotic disease was noted within other organs or tissues. Minimal to mild inflammation was present within the GI tract, kidneys, and heart. Lymph nodes exhibited mild to moderate lymphoid hyperplasia. |
| NC07 | No significant gross abnormalities. Changes within the right lower lobe are compatible with euthanasia artifact. | Pulmonary lesions in this animal included minimal to mild interstitial to perivascular inflammation within both the left and right lung lobes. One vessel within the right middle lung lobe was infiltrated by inflammatory cells consistent with vasculitis. The right lower lung lobe had scant foreign debris which was surrounded by minimal granulomatous inflammation. No significant lesions were noted within other tissues. Minimal to mild inflammation was present within the renal interstitial and GI tract. Lymph nodes (bronchial and mesenteric) exhibited lymphoid hyperplasia. Peripheral nodes had neutrophilic infiltration along the corticomedullary junction, the cause for the neutrophilic infiltration is unknown, but has been observed in animals on other projects and is therefore considered nonspecific. |

**Supplemental Table 4.** **Sampling schedule.**

| Specimen | Time point (dpi) | | | | | |
| --- | --- | --- | --- | --- | --- | --- |
|  | -7  (Baseline) | 1 | 7 | 14 | 21 | 28  (Necropsy) |
| Nasal swab (n) | 16 | 16 | 16 | 16 | 0 | 16 |
| Buccal swab (n) | 16 | 16 | 16 | 16 | 0 | 16 |
| Pharyngeal swab (n) | 16 | 16 | 16 | 16 | 0 | 16 |
| Rectal swab (n) | 16 | 16 | 16 | 16 | 0 | 16 |
| Blood (n) | 16 | 16 | 16 | 16 | 16 | 16 |
| Exhaled breath (n) | 16 | 16 | 16 | 16 | 16 | 16 |

n, the number of NHPs that received the sample collection.

**Supplemental Table 5. The sensitivity of RT-qPCR and CRISPR-FDS.**

|  |  | Swab samples | | | |  |  | |
| --- | --- | --- | --- | --- | --- | --- | --- | --- |
| Method | DPI | Nasal | Buccal | Pharyngeal | Rectal | Plasma | | Breath |
| RT-qPCR | 0 | 0.0% | 0.0% | 0.0% | 0.0% | 0.0% | | 0.0% |
|  | 1 | 100.0% | 37.5% | 100.0% | 18.8% | 6.3% | | 0.0% |
|  | 7 | 75.0% | 0.0% | 87.5% | 37.5% | 6.3% | | 0.0% |
|  | 14 | 100.0% | 0.0% | 37.5% | 43.8% | 0.0% | | 0.0% |
|  | 21 | -- | -- | -- | -- | 0.0% | | 0.0% |
|  | 28 | 50.0% | 0.0% | 0.0% | 6.3% | 0.0% | | 0.0% |
| CRISPR-FDS | 0 | 0.0% | 0.0% | 0.0% | 0.0% | 0.0% | | 0.0% |
|  | 1 | 100.0% | 93.8% | 50.0% | 50.0% | 50.0% | | 18.8% |
|  | 7 | 100.0% | 9.4% | 100.0% | 75.0% | 87.5% | | 37.5% |
|  | 14 | 100.0% | 93.8% | 81.3% | 75.0% | 100.0% | | 31.4% |
|  | 21 | -- | -- | -- | -- | 100.0% | | 25%% |
|  | 28 | 81.3% | 56.3% | 56.3% | 68.8% | 100.0% | | 12.5% |

**Supplemental Table 6. The sensitivity of RT-qPCR and CRISPR-FDS in different subgroup.**

| Specimen | DPI | CRISPR-FDS | | | |  | RT-qPCR | | | |
| --- | --- | --- | --- | --- | --- | --- | --- | --- | --- | --- |
|  |  | 1×10^4^ TCID_50_ | | 1.2×10^6^ TCID_50_ | |  | 1×10^4^ TCID_50_ | | 1.2×10^6^ TCID_50_ | |
|  |  | AGM  Aerosol | Rhesus  Aerosol | AGM  Multi-route | Rhesus  Multi-route |  | AGM  Aerosol | Rhesus  Aerosol | AGM  Multi-route | Rhesus  Multi-route |
| Nasal | 1 | 100% | 100% | 100% | 100% |  | 100% | 100% | 100% | 100% |
|  | 7 | 100% | 100% | 100% | 100% |  | 50% | 50% | 100% | 100% |
|  | 14 | 100% | 100% | 100% | 100% |  | 100% | 100% | 100% | 100% |
|  | 28 | 100% | 100% | 75% | 75% |  | 50% | 0% | 75% | 75% |
| Buccal | 1 | 100% | 75% | 100% | 100% |  | 25% | 50% | 25% | 50% |
|  | 7 | 100% | 75% | 100% | 100% |  | 0% | 0% | 0% | 0% |
|  | 14 | 100% | 75% | 100% | 100% |  | 0% | 0% | 0% | 0% |
|  | 28 | 25% | 50% | 75% | 75% |  | 0% | 0% | 0% | 0% |
| Pharyngeal | 1 | 50% | 0% | 75% | 75% |  | 100% | 100% | 100% | 100% |
|  | 7 | 100% | 100% | 100% | 100% |  | 100% | 100% | 50% | 100% |
|  | 14 | 100% | 75% | 100% | 50% |  | 75% | 25% | 0% | 50% |
|  | 28 | 25% | 75% | 100% | 25% |  | 0% | 0% | 0% | 0% |
| Rectal | 1 | 50% | 50% | 25% | 75% |  | 25% | 0% | 25% | 100% |
|  | 7 | 75% | 75% | 75% | 75% |  | 25% | 25% | 100% | 0% |
|  | 14 | 100% | 100% | 50% | 50% |  | 75% | 0% | 75% | 0% |
|  | 28 | 100% | 100% | 50% | 25% |  | 25% | 0% | 0% | 0% |
| Plasma | 1 | 100% | 75% | 0% | 25% |  | 25% | 0% | 0% | 0% |
|  | 7 | 100% | 100% | 100% | 50% |  | 0% | 25% | 0% | 0% |
|  | 14 | 100% | 100% | 100% | 100% |  | 0% | 0% | 0% | 0% |
|  | 21 | 100% | 100% | 100% | 100% |  | 0% | 0% | 0% | 0% |
|  | 28 | 100% | 100% | 100% | 100% |  | 0% | 0% | 0% | 0% |
| Breath | 1 | 25% | 25% | 25% | 0% |  | 0% | 0% | 0% | 0% |
|  | 7 | 75% | 25% | 50% | 0% |  | 0% | 0% | 0% | 0% |
|  | 14 | 50% | 25% | 50% | 0% |  | 0% | 0% | 0% | 0% |
|  | 21 | 25% | 25% | 50% | 0% |  | 0% | 0% | 0% | 0% |
|  | 28 | 0% | 25% | 25% | 0% |  | 0% | 0% | 0% | 0% |

**
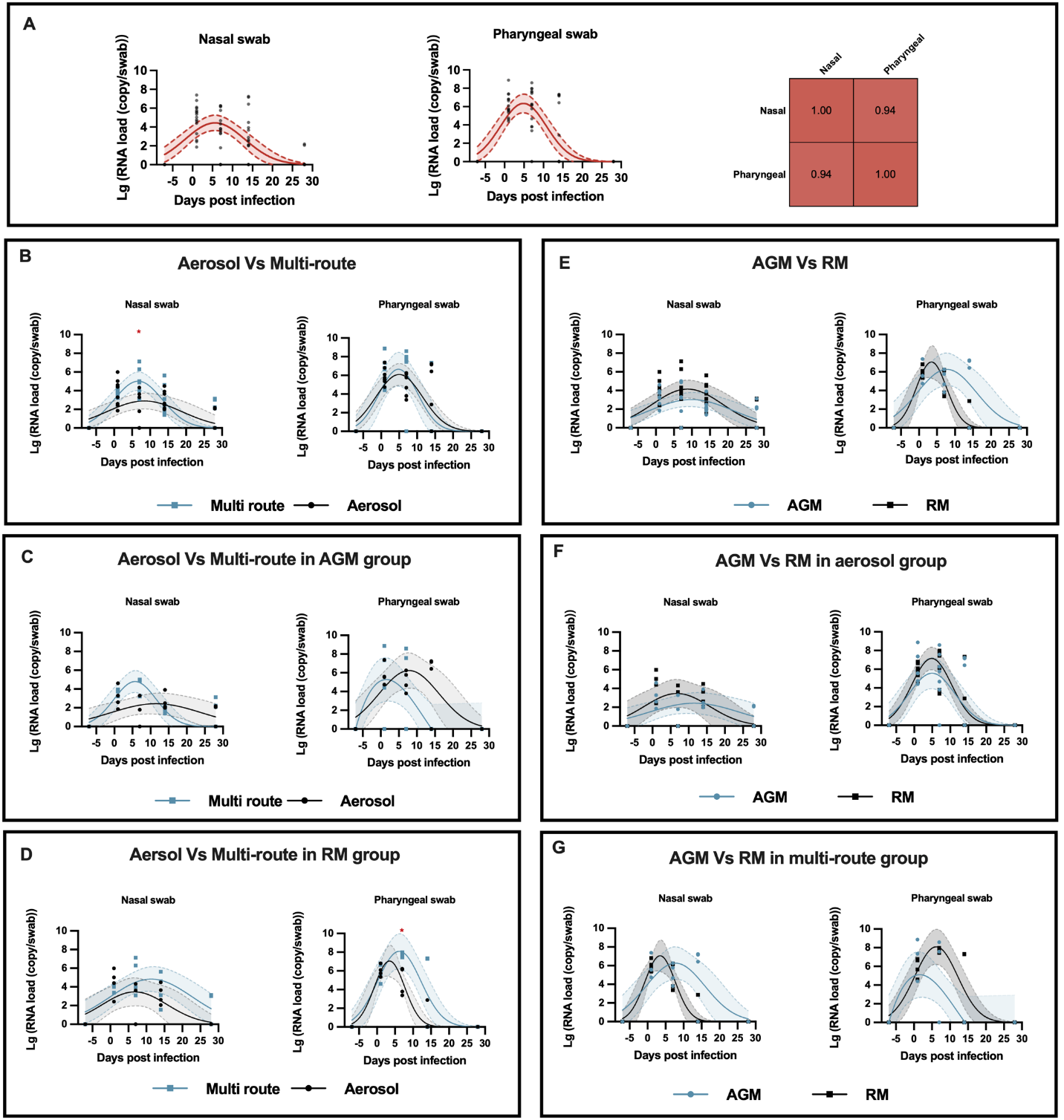
**

**Supplemental Fig. 1.** RT-qPCR SARS-CoV-2 signal in nasal and pharyngeal swab samples. (**A**) RT-qPCR signal for nasal and pharyngeal swab samples aggregated without respect to species or exposure route, displaying the correlation between longitudinal samples of each type. (**B-G**) RT-qPCR signal for nasal and pharyngeal swab samples by (**B-D**) virus exposure route, in aggregate or split by NHP species, or by (**E-G**) NHP species, in aggregate or split by virus exposure route. Data points represent the mean of technical replicates for each sample. Red, blue, and grey shaded regions and dashed lines indicate the 95% confidence intervals of the fitted lines. (*, P<0.05 by Mann-Whitney test)

**
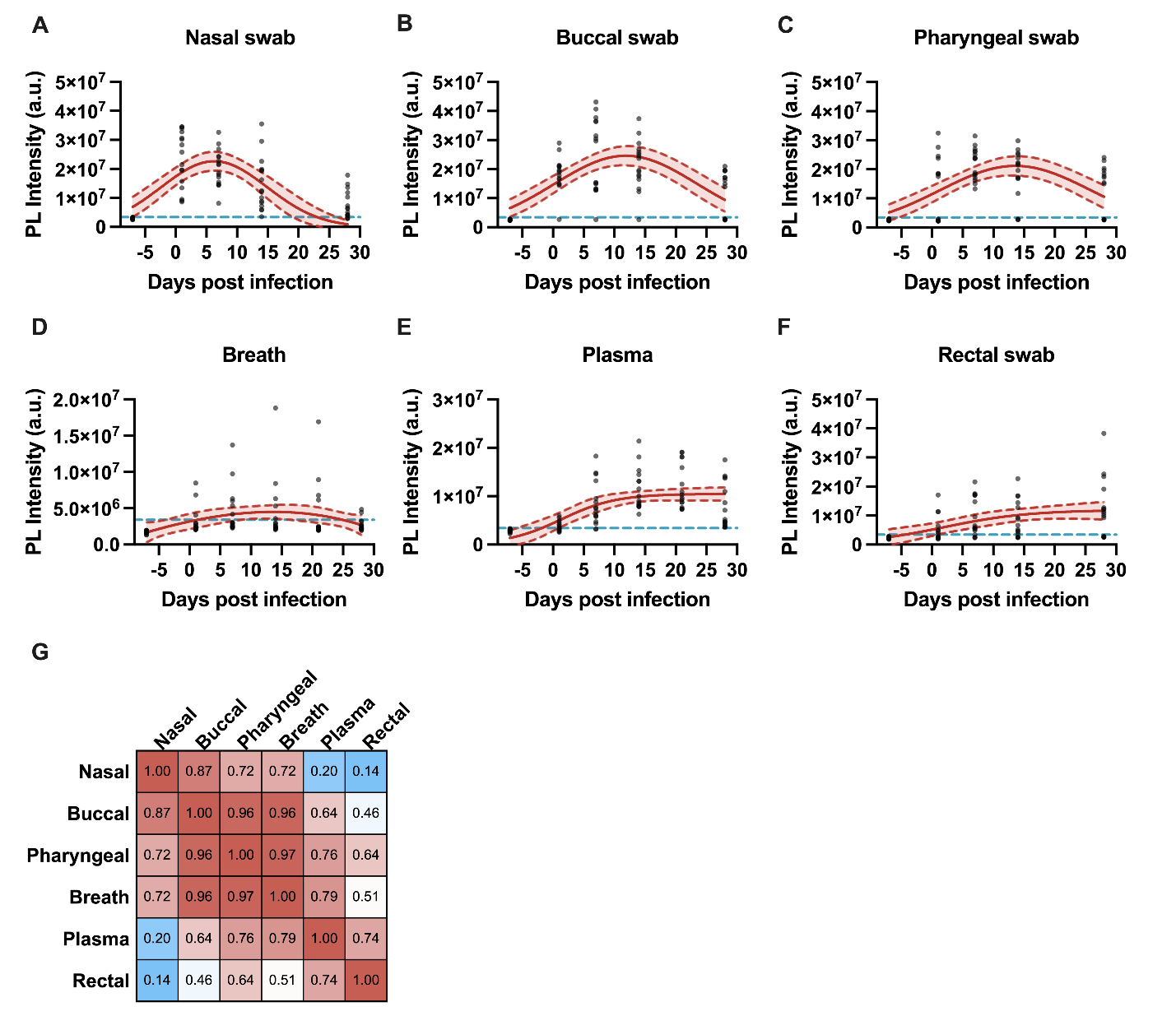
**

**Supplemental Fig. 2.** CRISPR-FDS SARS-CoV-2 signal in (**A**) nasal, (**B**) buccal, (**C**) pharyngeal swabs, (**D**) breath sample, (**E**) plasma, and (**F**) rectal swabs at the indicated time points, and (**G**) correlation between longitudinal signals detected for each of these sample types. Data points represent the mean of technical replicates for each sample. Red shaded regions and dashed lines indicate the 95% confidence intervals of the fitted lines. Blue dashed lines indicate the threshold for positive CRISPR-FDS signal (3.4 × 10^6^ a.u.) defined as the mean plus 3 times the standard deviation of the CRISPR-FDS signal detected in baseline samples. Correlation matrix colors denote the strength of the Pearson correlation coefficient (r-value) for each comparison from strong (red) to weak (blue).


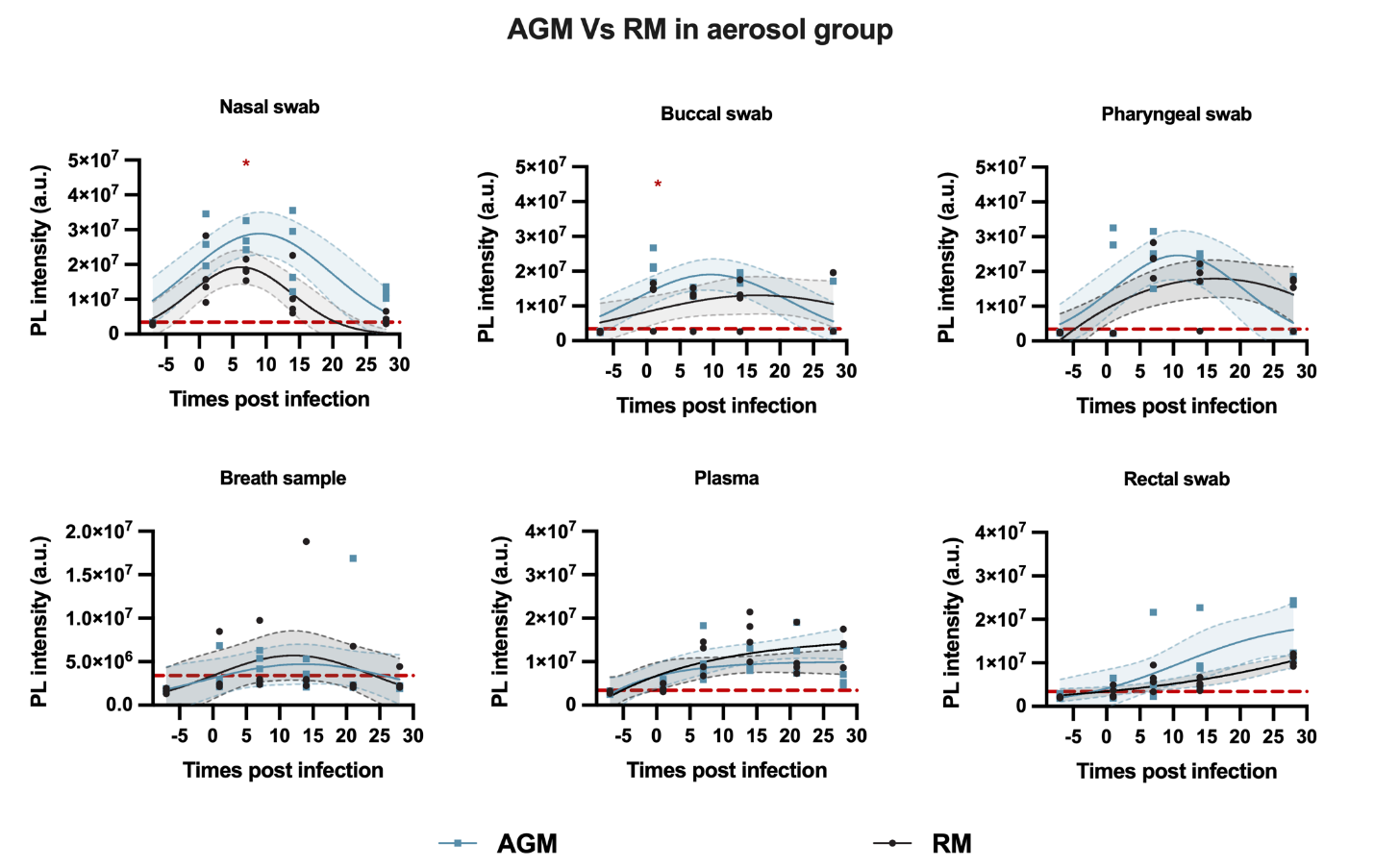
 **Supplemental Fig. 3.** CRISPR-FDS SARS-CoV-2 signal in African green monkeys (AGM, n=4) and Indian Rhesus macaques (RM, n=4) infected by aerosol exposure. Data points represent the mean of technical replicates for each sample. Blue and grey shaded regions and dashed lines indicate the 95% confidence intervals of the fitted lines. Red dashed lines indicate the threshold for positive CRISPR-FDS signal (3.4 × 10^6^ a.u.) defined as the mean plus 3 times the standard deviation of the CRISPR-FDS signal detected in baseline samples. (*, P<0.05 by Mann-Whitney test)

**
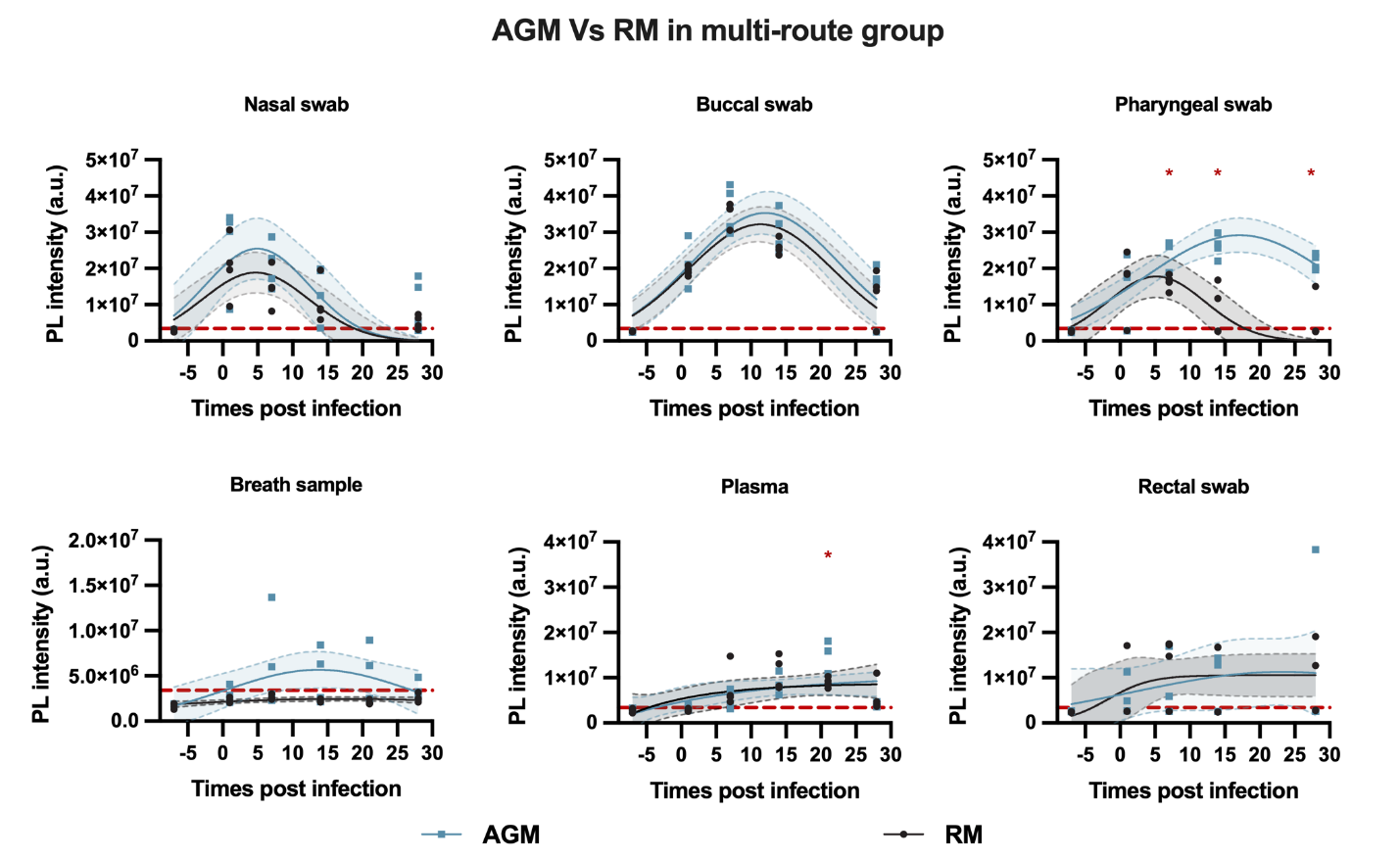
** **Supplemental Fig. 4.** CRISPR-FDS SARS-CoV-2 signal in African green monkeys (AGM, n=4) and Indian Rhesus macaques (RM, n=4) infected by multi-route exposure. Data points represent the mean of technical replicates for each sample. Blue and grey shaded regions and dashed lines indicate the 95% confidence intervals of the fitted lines. Red dashed lines indicate the threshold for positive CRISPR-FDS signal (3.4 × 10^6^ a.u.) defined as the mean plus 3 times the standard deviation of the CRISPR-FDS signal detected in baseline samples. (*, P<0.05 by Mann-Whitney test)

**
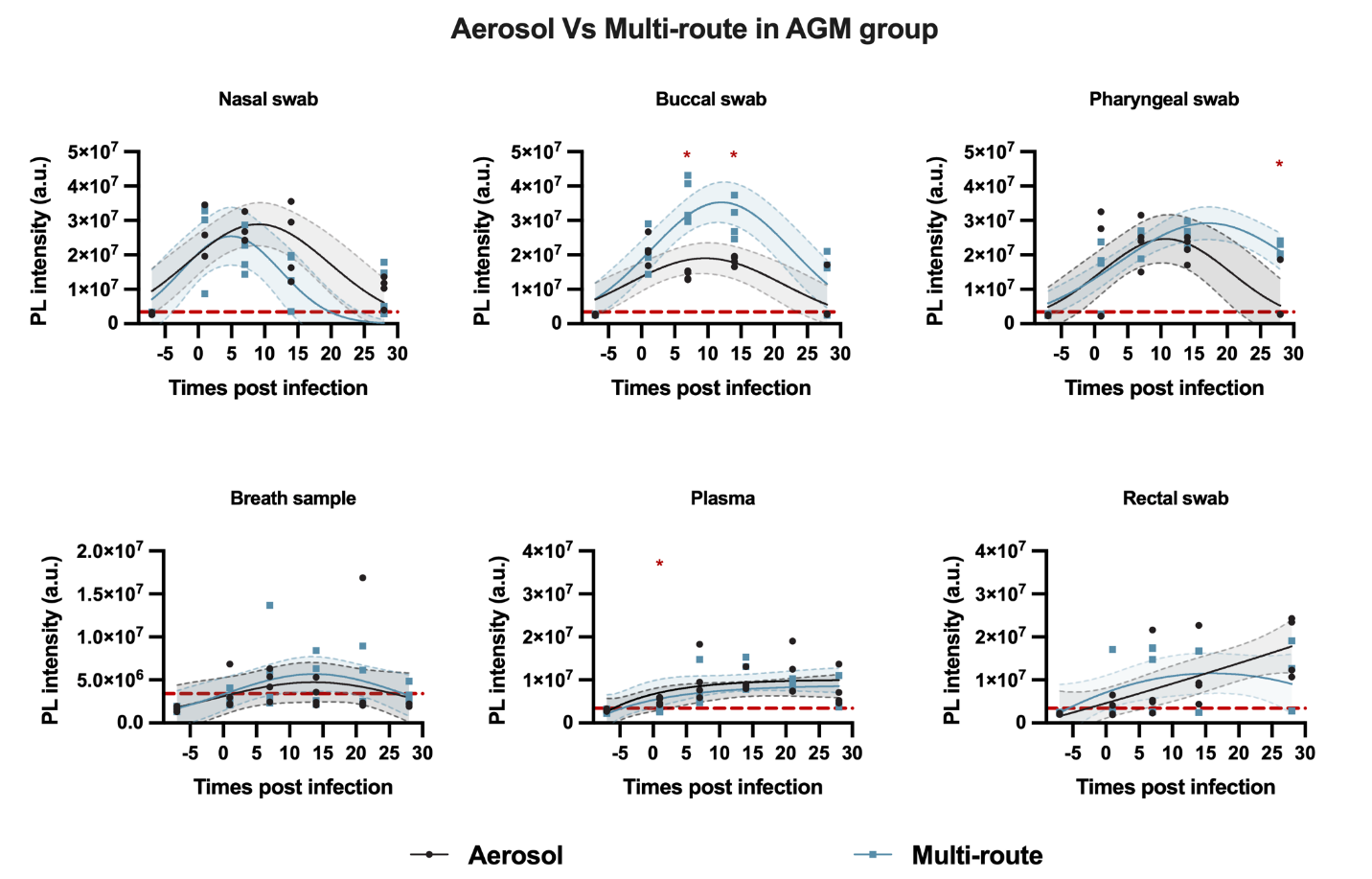
****Supplemental Fig. 5.** CRISPR-FDS SARS-CoV-2 signal in African green monkeys infected by aerosol (n=4) or multi-route (n=4) exposure. Data points represent the mean of technical replicates for each sample. Blue and grey shaded regions and dashed lines indicate the 95% confidence intervals of the fitted lines. Red dashed lines indicate the threshold for positive CRISPR-FDS signal (3.4 × 10^6^ a.u.) defined as the mean plus 3 times the standard deviation of the CRISPR-FDS signal detected in baseline samples. (*, P<0.05 by Mann-Whitney test)


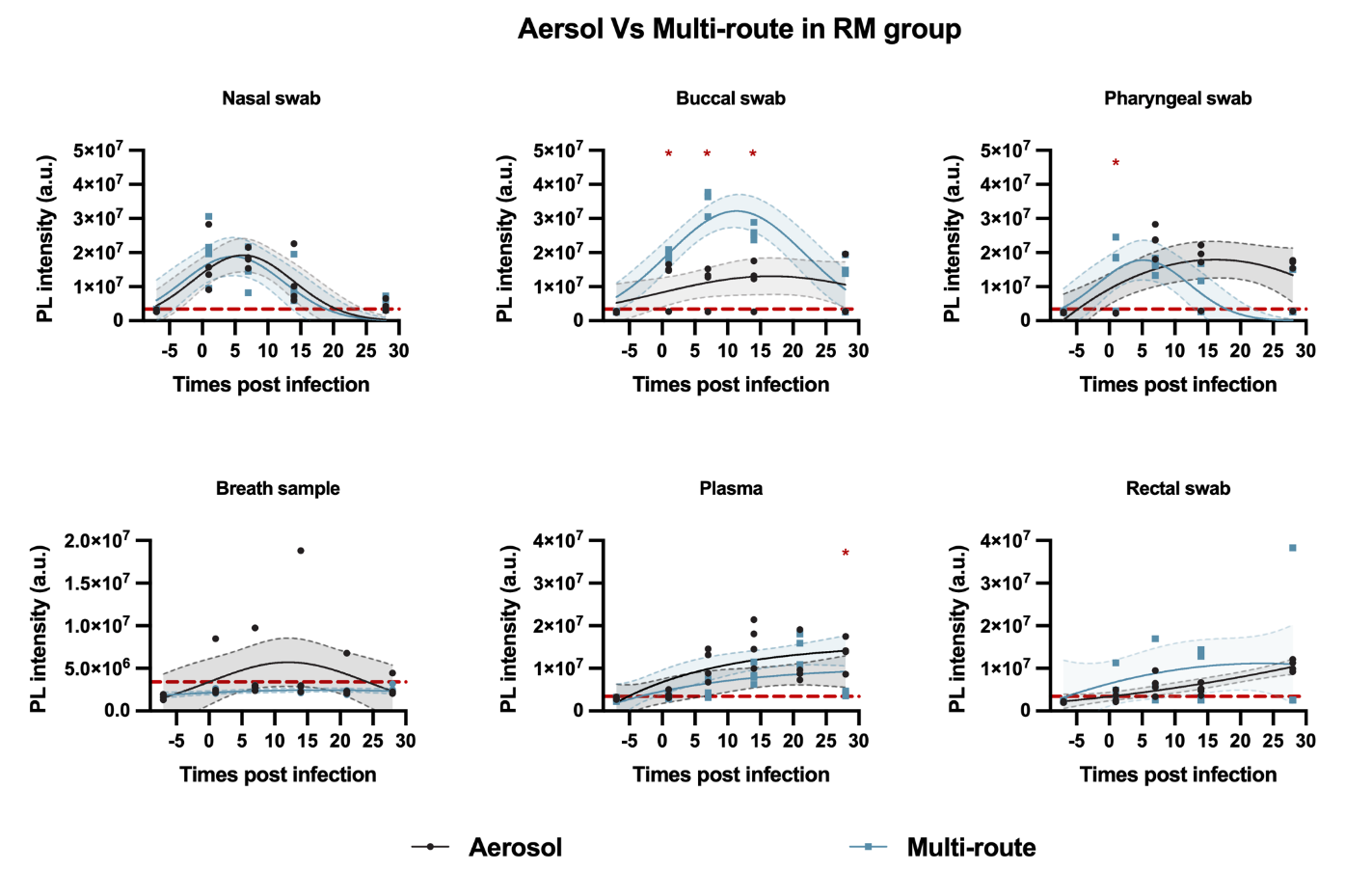


**Supplemental Fig. 6.** CRISPR-FDS SARS-CoV-2 signal in Indian Rhesus macaques infected by aerosol (n=4) or multi-route (n=4) exposure. Data points represent the mean of technical replicates for each sample. Blue and grey shaded regions and dashed lines indicate the 95% confidence intervals of the fitted lines. Red dashed lines indicate the threshold for positive CRISPR-FDS signal (3.4 × 10^6^ a.u.) defined as the mean plus 3 times the standard deviation of the CRISPR-FDS signal detected in baseline samples. (*, P<0.05 by Mann-Whitney test)

**Supplemental Fig. 7**. Case history for Case-1 shown in Figure 4: a 54-year-old female with pre-existing acute lymphoblastic leukemia, had multiple negative RT-qPCR results with respiratory samples obtained following chest radiography results suggestive of COVID-19, and was therefore started on antibiotic therapy (ABT) for pneumonia and administered intravenous immunoglobulin for hypogammaglobulinemia. Over the following 48 hours her respiratory status worsened to require supplemental oxygen, at which time multiple CRISPR-FDS test returned results positive for SARS-CoV-2. This patient was then transferred to the COVID-19 isolation ward on suspicion of an active SARS-CoV-2 infection, received a unit of COVID-19 convalescent plasma therapy (CCPT), and exhibited marked reductions in her shortness of breath, cough, fever, and supplemental oxygen requirement within the next 24 hours. Due to continual lingering symptoms, this patient received an additional unit of CCP 1 week after initial infusion, after which she continued to improve and was discharged with resolution of all symptoms.

**Supplemental Fig. 8.** Case histories for Cases-2 and -3 shown in Figure 4. Case-2, a 37-year-old female exhibited severe symptoms (fatigue, myalgia, fever, cough, shortness of breath, and headache) symptom onset tested negative for COVID-19 by a nasal swab RT-qPCR tests performed the following day and IgM positive at day two after symptom onset, suggesting this individual had an extended latency period between her initial exposure and symptoms onset. Case-3, a 38-year-old male who was a close contact of Case-2, developed minimal symptoms one day after Case-3 and did not receive a nasal swab RT-qPCR test for COVID-19. However, both Case-2 and -3 had tested positive for SARS-CoV-2 RNA in nasal swab, plasma, and saliva samples collected at 2 days after their respective symptom onsets.

**Supplemental Fig. 9.** Case history for Case-4 shown in Figure 4: a 64-year-old male, with history of T-cell ALL, was initially admitted for scheduled chemotherapy for his T Cell-ALL. Upon admission, patient tested negative for SARS-CoV-2 RNA by nasopharyngeal RT-qPCR, and received his chemotherapy regimen for 4 days without issue, but he acutely developed dyspnea and hypoxia on hospital day 5 (his anticipated discharge date), and was transferred to the ICU. A second nasopharyngeal RT-qPCR performed at this time was negative, but a CT image revealed an increase in bilateral airspace and ground glass opacities. He was treated with broad spectrum antibiotics (ABT) for possible pneumonia, and continued to require supplemental O_2_ despite aggressive antibiotics and diuresis. A CRISPR-FDS test conducted on hospital day 8 detected SARS-CoV-2 RNA in a nasal swab, but not a plasma sample. The patient received one unit of COVID-19 convalescent plasma therapy (CCPT) on hospital day 9, after which his oxygenation status improved significantly, although he still intermittently required supplemental O_2_ when ambulating. He received an additional CCP on day 16, continued to improve and was discharged on hospital day 20.

**Supplemental Fig.** **10.** Cases history for Case-5 shown in Figure 4: a 26-year-old female had a history of AML and presented with fever, tachycardia and hypotension, and a right upper lung lobe nodule, but tested negative for COVID-19 by nasal swab RT-qPCR. She was started on broad spectrum antibiotics (ABT) and antifungals, but continued to spike fevers with tachycardia and hypoxia, and a second CT revealed bilateral diffuse ground glass opacity within the lower lung. A second nasal swab RT-qPCR test for COVID-19 performed on hospital day 6 was negative. She was continued on broad spectrum antibiotics (ABT) with an escalated antifungal treatment, after which she slowly improved, and was discharged for treatment of her AML at another site. Due to the absence RT-qPCR positive results and a positive response to antibiotics, this patient was considered not to had COVID-19. Retrospective CRISPR-FDS analysis of stored nasal swab and plasma samples from this patient were negative for SARS-CoV-2 RNA.

**Supplemental Fig. 11.** Case history for Case-6 shown in Figure 4: a 24-year-old male initially presented to an outside hospital with concern for a new diagnosis of acute myeloid leukemia (AML), and was noted to have had intermittent flu-like symptoms for roughly 3 months prior to admission, at which time he was found to have a moderate sized pericardial effusion and cardiac tamponade, and a pericardial window and drain were placed roughly 2 days prior to transfer to Tulane Medical Center to relieve tamponade physiology. He was also diagnosed with a post-obstructive pneumonia, for which he was started on broad spectrum antibiotics prior to transfer. Bilateral pleural effusion noted for this case after transfer was considered likely to have arisen from tamponade physiology, but right upper lobe ground glass opacities noted on the chest CT in this patient appeared consistent with COVID-19, and were judged by the attending infectious disease physicians to be less likely to represent atypical pneumonia. This patient had negative nasal RT-PCR test results at admission and upon transfer, and worsened following antibiotic therapy (ABT), becoming tachycardiac and hypotensive, demonstrated increased respiratory effort, and was transferred to the ICU where he was started on a broader course of antibiotics without major signs of improvement. Workups for other infectious etiologies all came back negative, and the patient again tested negative for COVID-19 by nasal swab RT-qPCR, but tested positive upon investigational use of a CRISPR-FDS assay, and improved upon subsequent treatment with COVID-19 convalescent plasma therapy (CCPT) and chemotherapy.

**Supplemental Fig. 12.** Case history for Case-7 shown in Figure 4: a 35-year-old male with history of classic Hodgkin’s Lymphoma on chemotherapy, who presented with 1.5 weeks of shortness of breath, chest pain, neck pain, and headache. Two nasal swab RT-qPCR test for COVID-19 both returned negative results, and a chest CT chest revealed no clear finding of consolidation or ground-glass opacities, but did reveal an enlarged mediastinal mass concerning for possible disease recurrence. Investigational CRISPR-FDS tests performed on nasal swab and plasma samples from this patient both tested positive for SARS-CoV-2 RNA. She remained clinically stable, however, and was subsequently discharged without having received any specific treatment for COVID-19.
